# The microenvironment determines the breast cancer cells' phenotype: organization of MCF7 cells in 3D cultures

**DOI:** 10.1186/1471-2407-10-263

**Published:** 2010-06-07

**Authors:** Silva Krause, Maricel V Maffini, Ana M Soto, Carlos Sonnenschein

**Affiliations:** 1 Sackler School of Graduate Biomedical Sciences, 136 Harrison Avenue, Tufts University School of Medicine, Boston, MA, USA; 2 Department of Anatomy and Cellular Biology, 136 Harrison Avenue, Tufts University School of Medicine, Boston, MA, USA; 3 Current Address: Children's Hospital Boston and Harvard Medical School, Vascular Biology Program, Department of Surgery, Boston, MA, USA

## Abstract

**Background:**

Stromal-epithelial interactions mediate breast development, and the initiation and progression of breast cancer. In the present study, we developed 3-dimensional (3D) in vitro models to study breast cancer tissue organization and the role of the microenvironment in phenotypic determination.

**Methods:**

The human breast cancer MCF7 cells were grown alone or co-cultured with primary human breast fibroblasts. Cells were embedded in matrices containing either type I collagen or a combination of reconstituted basement membrane proteins and type I collagen. The cultures were carried out for up to 6 weeks. For every time point (1-6 weeks), the gels were fixed and processed for histology, and whole-mounted for confocal microscopy evaluation. The epithelial structures were characterized utilizing immunohistochemical techniques; their area and proliferation index were measured using computerized morphometric analysis. Statistical differences between groups were analyzed by ANOVA, Dunnett's T3 post-hoc test and chi-square.

**Results:**

Most of the MCF7 cells grown alone within a collagen matrix died during the first two weeks; those that survived organized into large, round and solid clusters. The presence of fibroblasts in collagen gels reduced MCF7 cell death, induced cell polarity, and the formation of round and elongated epithelial structures containing a lumen. The addition of reconstituted basement membrane to collagen gels by itself had also survival and organizational effects on the MCF7 cells. Regardless of the presence of fibroblasts, the MCF7 cells both polarized and formed a lumen. The addition of fibroblasts to the gel containing reconstituted basement membrane and collagen induced the formation of elongated structures.

**Conclusions:**

Our results indicate that a matrix containing both type I collagen and reconstituted basement membrane, and the presence of normal breast fibroblasts constitute the minimal permissive microenvironment to induce near-complete tumor phenotype reversion. These human breast 3D tissue morphogenesis models promise to become reliable tools for studying tissue interactions, therapeutic screening and drug target validation.

## Background

Stromal-epithelial interactions play important roles during mammary gland development. This intense epithelial-mesenchymal crosstalk has been well documented during the process of mammary bud invasion of the underlying mesenchyme [1,2]. Comparable reciprocal interactions are also evident during tumor initiation and progression as highlighted in tissue recombination experiments. By recombining stroma and epithelium under different experimental conditions it was demonstrated that normal mammary epithelial cells were "instructed" to become tumor cells only when the stroma was treated with a carcinogen [3,4]. Moreover, transgenic mice that over-expressed the extracellular matrix (ECM)-degrading enzyme stromelysin-1 (also known as matrix metalloproteinase 3 or MMP3) in the mammary stroma, developed mammary epithelial tumors at approximately 3-4 months of age [5,6]. Equally significant, normal stroma induced epithelial tumor cells to form normal mammary ducts [7]. Furthermore, mouse mammary tumor cells cultured *in vitro *with embryonic mouse mammary mesenchyme normalized their phenotype and formed mammary ducts [8]. These phenotypic reversions have also been documented in the liver where hepatocarcinoma cells acquired normal liver cell phenotypes when injected into a healthy rat hepatic parenchyma [9,10].

Changes in the stroma have been reported to accompany cancer progression leading to metastases [11-13]. Such stromal changes include the induction or upregulation of a variety of molecules such as growth factors, matrix degrading enzymes, angiogenic factors and cytokines in stromal cells [14]. In addition, tumor-derived fibroblasts produced a collagen-rich matrix [15]. Using an animal model, Provenzano *et al*. observed that increased collagen density promoted mammary tumor initiation and progression [16].

Because of the complexity of *in vivo *experiments, three-dimensional (3D) cell cultures are being increasingly used as surrogate models in order to explain how cell-matrix interactions affect the morphology, differentiation, proliferation, and apoptosis of breast cancer epithelial cells. Whereas cell morphology of normal and breast cancer epithelial cells is similar in 2D, non-malignant and malignant cells can be reliably distinguished when grown in 3D reconstituted basement membrane (rBM) (commonly known as Matrigel) cultures [17]. While non-malignant cells organize into polarized, growth-arrested colonies, malignant cells - both established and primary tumor cells - show a loss of tissue polarity, a disorganized architecture without lumen and failure to arrest growth [17-20]. Furthermore, sensitivity to chemotherapeutic agents dramatically decreased in 3D cultures compared to the effects shown by the same drugs in 2D [21-24]; thus 3D cultures appear to be better models for the cytotoxic evaluation of anticancer drugs [25].

Breast cancer cells derived from either established cell lines or from primary tumors formed disorganized cell clusters without lumen when grown in rBM [17-19]. A comparison between the morphology and gene expression profile of several breast cancer cell lines grown in 3D cultures using rBM as a matrix showed that cells expressing similar phenotypes clustered together based on similarities in their gene expression [18]. The commonly used human breast cancer MCF7 cell line formed round colonies with disorganized nuclei and absent lumen when cultured for 4 days using only rBM as the matrix. In addition to rBM and collagen gels, polylactic acid (PLA) polymers, chitosan scaffolds and poly(lactide-co-glycolide) (PLG) scaffolds have been shown to support growth of MCF7 cells [21,25,26]. Recently, a 3D mammalian cell perfusion-culture system using microfluidic channels was shown to support 3D cell-cell and cell-matrix interactions using MCF7 cells [27]. Although much is known about the important role of the stroma in determining the epithelial phenotype, research continues to be focused mostly on the epithelial cells while disregarding the influence of their microenvironment. Few papers have reported the use of co-cultures of breast cancer cells with other cells found in the *in vivo *tumor microenvironment including adipocytes [28] and osteoblasts found in metastatic sites [29].

Herein, we report the role of two stromal components, namely human breast fibroblasts, and ECM composition in determining the morphology, survival and differentiation of the human breast cancer MCF7 cell line in a long-term 3D tissue morphogenesis model.

## Methods

### Chemicals and cell culture reagents

Methyl salicylate, Mayer's hematoxylin, HEPES and Carmine Alum were purchased from Sigma-Aldrich (St. Louis, MO). Dulbecco's modified Eagle's medium (DMEM) was purchased from MP Biomedicals (Solon, OH). DMEM/F12 and penicillin-streptomycin solution were obtained from Gibco/Invitrogen (Carlsbad, CA). Fetal bovine serum (FBS) was purchased from HyClone (Logan, UT). Bovine type I collagen was purchased from Organogenesis (Canton, MA). Matrigel™ and rat tail type I collagen were purchased from BD Biosciences (San Jose, CA). Formalin was obtained from Fisher Scientific (Atlanta, GA).

### Cell maintenance

All cells were maintained and expanded in cell culture plastic flasks (Corning, Corning, NY). MCF7 cells were grown in DMEM containing 5% FBS. Human mammary fibroblasts obtained from reduction mammoplasties (RMF) were purchased from ScienCell (Carlsbad, CA). RMF were routinely grown in DMEM containing 10% FBS, 15 μM HEPES and penicillin-streptomycin. All cells were incubated at 37°C and 6% CO_2_. For co-culture experiments, a combined medium (1 part of MCF7 medium and 1 part of RMF medium) was used. The combined medium was tested in tissue culture flasks containing each cell type alone, i.e. either MCF7 cells or RMF, to assure proper growth and behavior of cells.

### 3D multicellular culture

Type I collagen was used at a concentration of 1 mg/ml according to Paszek et al. [30]. Collagen was neutralized according to the manufacturer's instructions. Collagen gels were prepared using bovine collagen; mixed Matrigel™ and collagen gels were prepared using a 1:1 volume ratio of Matrigel™ and type I collagen keeping the final collagen concentration at 1 mg/ml. In co-cultures, 300,000 MCF7 cells and 100,000 RMF were used to mimic the *in vivo *ratio of epithelial cells to fibroblasts in the human breast [31]. The same number of cells was seeded for each cell type cultured independently. Cells were suspended in 3 ml collagen or a 3 ml Matrigel™-collagen mixture and seeded into 35 mm well inserts of a six-well plate (Organogenesis, Canton, MA) as previously described [32]. The gels were allowed to solidify for 30 minutes at 37°C before adding combined medium onto each gel (2 ml) and into each well (10 ml). Cultures were maintained for one to six weeks and the medium was changed every two to three days.

### Gel processing

On the harvest day, the circular gels were cut into two pieces: one half was fixed overnight in 10% phosphate-buffered formalin, paraffin-embedded and used for histological analysis, and the second piece was whole-mounted onto a slide and fixed overnight in 10% phosphate-buffered formalin for morphometric analysis and confocal microscopy. The whole-mounted gels were stained with Carmine Alum overnight as described previously [33]. After staining, the whole mounts were progressively dehydrated in 70%, 95% and 100% ethanol, cleared in xylene and mounted with Permount™ (Fisher Scientific, Atlanta, GA).

### Confocal microscopy

Whole-mounted gels were analyzed using a Zeiss LSM 510 system (Carl Zeiss MicroImaging Inc, Thornwood, NY). The HeNE 633 nm/5 mW laser was used for data acquisition due to the autofluorescence of Carmine dye at this wavelength. The epithelial structures formed in collagen and mixed Matrigel™-collagen gels were scanned with a 20× objective lens, and 8 bit depth images with a resolution of up to 2048 × 2048 pixels were taken. The data were three-dimensionally reconstructed using Zeiss software.

### Immunohistochemical analysis

All primary antibody concentrations and supplier information are listed in Table 1. Apoptotic cells were stained using a commercially available TUNEL staining kit (Roche Applied Science, Indianapolis, IN). An antigen-retrieval method using microwave pretreatment and 0.01 M sodium citrate buffer (pH 6) was used for all antibodies except laminin 5 [33]. The antigen-antibody reaction was visualized using the streptavidin-peroxidase complex, with diaminobenzidine tetrahydrochloride (Sigma-Aldrich, St. Louis, MO) as the chromogen. Counterstaining was performed with Mayer's hematoxylin. Images were captured using a Zeiss Axioscope 2 plus microscope (Carl Zeiss MicroImaging Inc, Thornwood, NY).

**Table 1:** List of primary antibodies used in immunohistochemical analyses.

Primary Antibody	Marker for:	Source	Dilution
Mouse anti keratin 18	Luminal epithelial cells	Sigma-Aldrich (St. Louis, MO)	1:500

Rabbit anti Ki67	Proliferation	Vector (Burlingame, CA)	1:3000

Mouse anti E-cadherin	Cell-cell adhesion	Novocastra (Newcastle, UK)	1:50

Mouse anti laminin 5	Basement membrane	Chemicon/Millipore (Bedford, MA)	1:200

Mouse anti sialomucin	Apical proteins	Abcam (Cambridge, MA)	1:800

Mouse anti-CEACAM	Cell-cell adhesion	Abcam (Cambridge, MA)	1:50

### Morphometric analysis

The area of the epithelial structures was measured at one, two and three weeks in culture. The analysis was done in both matrix conditions and in mono- and co-cultures. Three to four experiments for each condition were analyzed, and for each experiment, three arbitrarily chosen fields at 10× magnification per section were examined; two sections for each condition were used. There were between 10 and 20 colonies per field; the number of colonies per field decreases with time due to their increase in size. The sections were an accurate representation of the gel. Images were captured using a Zeiss AxioCam camera attached to a Zeiss Axioscope 2 plus using a 10× objective and 3900 dpi, and analyzed with the Zeiss Axiovision version 4.4 software (Carl Zeiss MicroImaging Inc, Thornwood, NY). The following parameters were measured per section: total number of structures, area of clusters, total number of cells per structure, and number of Ki67 positive cells within those structures. Proliferating epithelial cells were expressed as %Ki67 positive cells per total epithelial cell number. Morphometric analysis was carried out in collagen and mixed Matrigel™-collagen gels.

### Statistics

SPSS software package 15.0 (SPSS Inc., Chicago, IL) was used for all statistical analyses. ANOVA was used to compare the area of epithelial structures across time points within a single treatment. Dunnett's T3 post-hoc tests were used to determine which time points were significant within each individual treatment. All results are presented as mean ± s.e.m. Chi square tests were used to compare each condition in the Ki67 data, because proliferation index is expressed as a proportion. For all statistical tests, results were considered significant at p < 0.05.

## Results

### Breast morphogenesis in type I collagen gels

#### MCF7 cells in collagen gels

**Figure 1:**
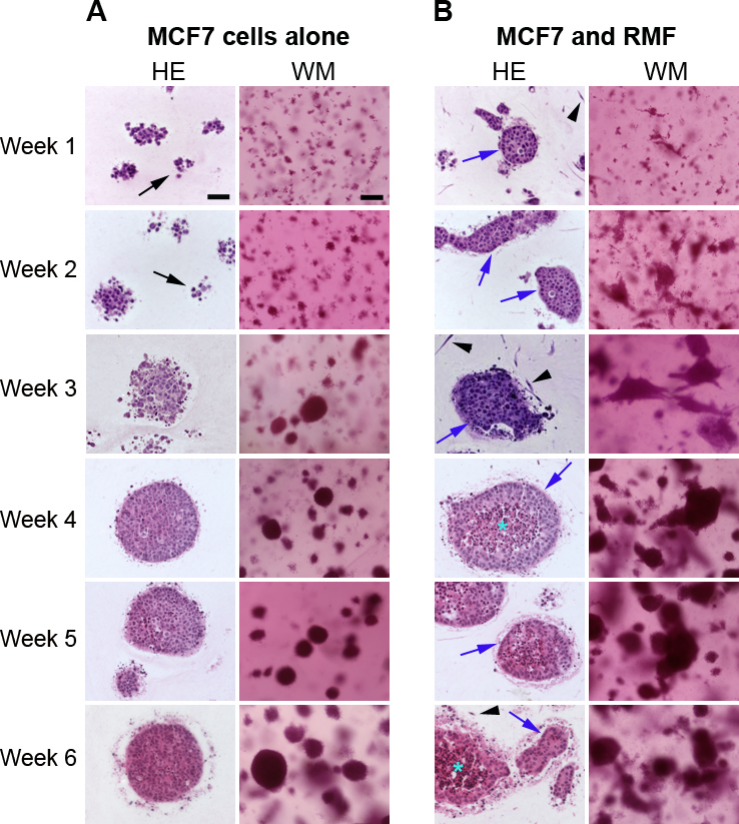
**Epithelial structures observed in collagen gels**. Panel A depicts representative H&E and whole mounts (WM) of MCF7 cells grown alone in collagen gels for 1-6 weeks. Dead cells are depicted by black arrows. Panel B shows MCF7 cells grown in co-culture with RMF in collagen gels. Note the survival effect provided by the presence of RMF already at week 1. Arrowheads depict fibroblasts, blue arrows depict polarized structures, and * illustrates apoptotic cells. Scale bars: H&E = 50 μm, WM = 100 μm.

**Figure 2:**
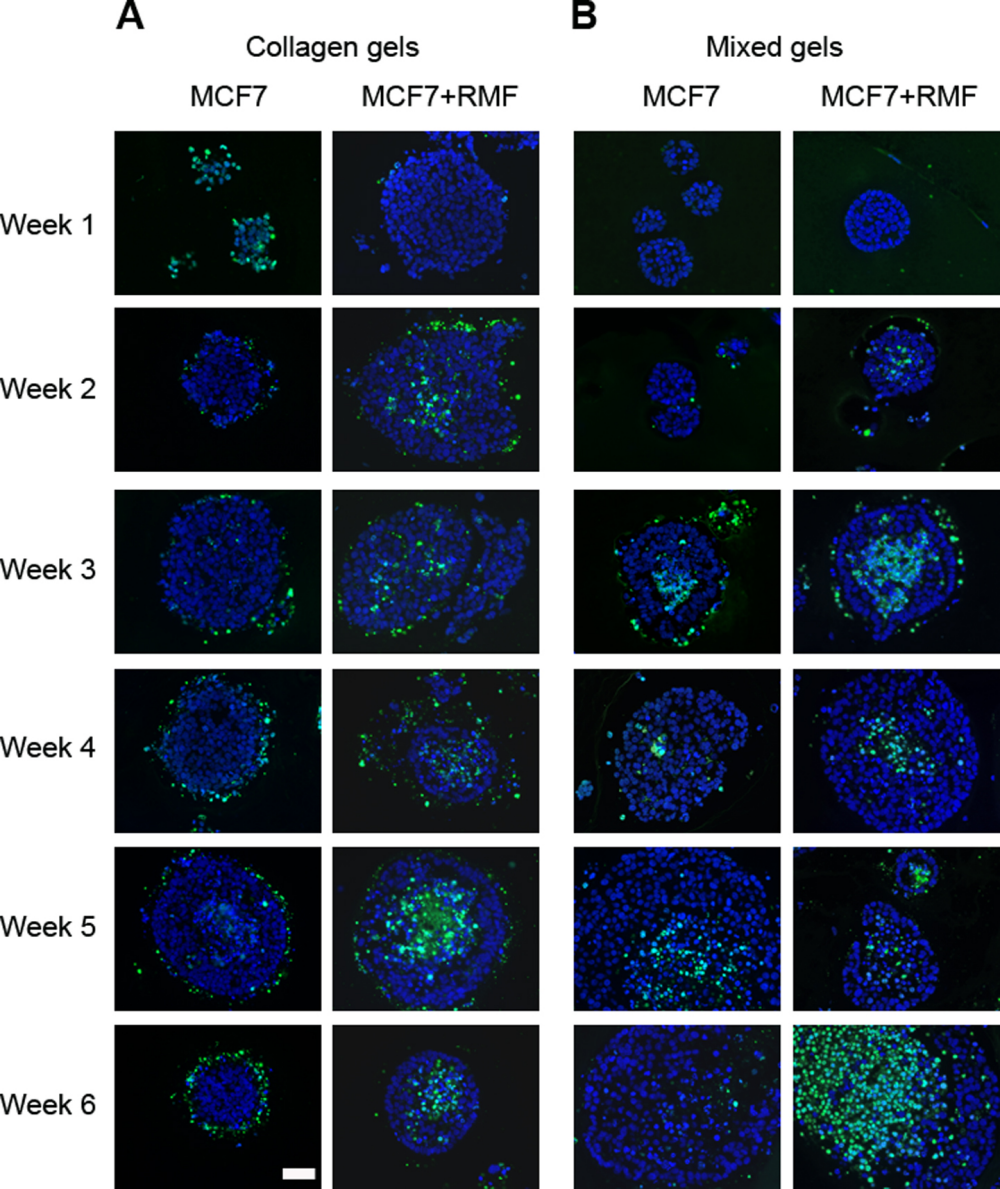
**Apoptosis in epithelial structures**. Cell death was assessed using the TUNEL assay. Blue: DAPI, Green: apoptotic cells. Scale bar: 50 μm

**Figure 3:**
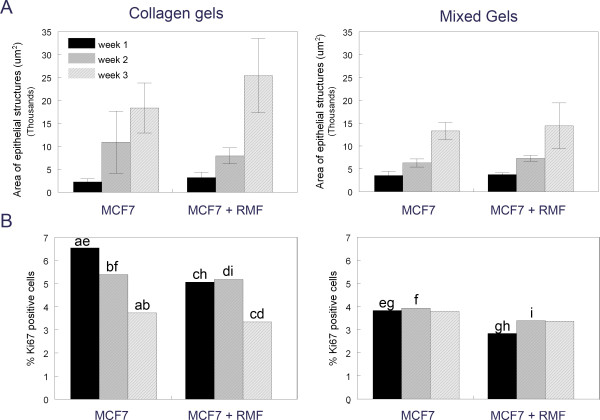
**Morphometric analysis of area and proliferation index of epithelial structures in type I collagen and mixed gels**. Panel A: The area was measured as μm^2^. Panel B: Proliferating cells are expressed as % Ki67 positive cells compared to all cells. Same letters denote significant differences between conditions and time points (p < 0.05).

Type I collagen is the major constituent of breast stroma and thus was utilized as a matrix for MCF7 cells grown in 3D cultures. Surprisingly, a large number of MCF7 cells died when cultured alone within these gels, and this was obvious during the first two weeks in culture. After two weeks in the 3D conditions, the surviving MCF7 cells formed rather loose cell clusters containing approximately 10-20 cells (Figure 1A, 2). These clusters increased in size over time; Figure 3A shows the increase in area of these epithelial clusters from week one to week three, although the difference was not statistically significant (p > 0.05). The epithelial structures continued to grow through week six (Figure 1). In addition, the proliferation index measured as percentage of cells expressing Ki67 protein decreased over time, the percentage of proliferating cells was significantly lower after three weeks compared to one and two weeks in culture (p < 0.05) (Figure 3B). The mean percentages of Ki67 positive cells were 6.55% (5.57-9.36%), 5.39% (4.23-9.15%), and 3.73% (2.73-5.3%) for weeks one, two and three, respectively (Figure 3B). MCF7 cells within the clusters did not polarize and no lumen was observed. Cell death in the center of these clusters was rarely observed despite the size of these structures (Figure 2A). In contrast, cell death was typically observed in the periphery of many clusters after three weeks and persisted throughout the six weeks in culture (Figures 1A, 2A).

#### Co-culture of MCF7 cells with RMF in collagen gels

The presence of RMF considerably improved the survival of MCF7 cells within collagen gels. After just one week in culture, tighter cell clusters with defined borders had formed (Figure 1B) and apoptotic cells were no longer observed (Figure 2A). Although apoptosis was observed at later time points, the degree and localization of apoptotic cells changed; in particular, 4-6 week-old cell clusters showed considerable apoptosis in the center of cell colonies. These epithelial structures significantly increased in size over time (p < 0.05) (Figure 3A). However, the addition of RMF did not significantly increase the area of the epithelial structures when compared to those formed by MCF7 cells grown alone (p > 0.05). These structures continued to grow through week six. The proliferation index remained similar between week one and two with means of 5.07% (4.12-6.63%) and 5.18% (3.64-9.36), respectively; by week three a significant decrease was observed with a mean of 3.34% (2.31-4.53%) compared to weeks one and two (p < 0.05) (Figure 3B). Importantly, after two weeks in culture with RMF, MCF7 cells started to polarize by shifting their nucleus to a basal position whereas polarization was not observed at any time point when MCF7 cells were grown alone in collagen gels. Cell polarization was observed in many clusters (Figure 1B). Beginning at two weeks in culture, cell death was observed in the center of these clusters while still surrounded by multiple layers of viable MCF7 cells (Figure 2A). After five and six weeks in culture, most of these clusters were composed of one to three layers of polarized and viable cells surrounding a center containing apoptotic cells, suggesting the formation of a lumen (Figure 2A). Notably, in the collagen gels containing MCF7 cells alone, apoptosis was rarely observed in the center of clusters although these were of the same size as the structures observed in the co-cultures. As described for the MCF7 mono-cultures, dead cells were observed, even though to a lesser extent, in the periphery of most epithelial clusters, a phenomenon that began after two weeks and persisted throughout the six weeks in culture (Figure 2A). RMF grown alone in the collagen gels were homogenously distributed throughout the gel and continued to proliferate throughout the length of the experiments (data not shown).

#### Characterization of epithelial structures

**Figure 4:**
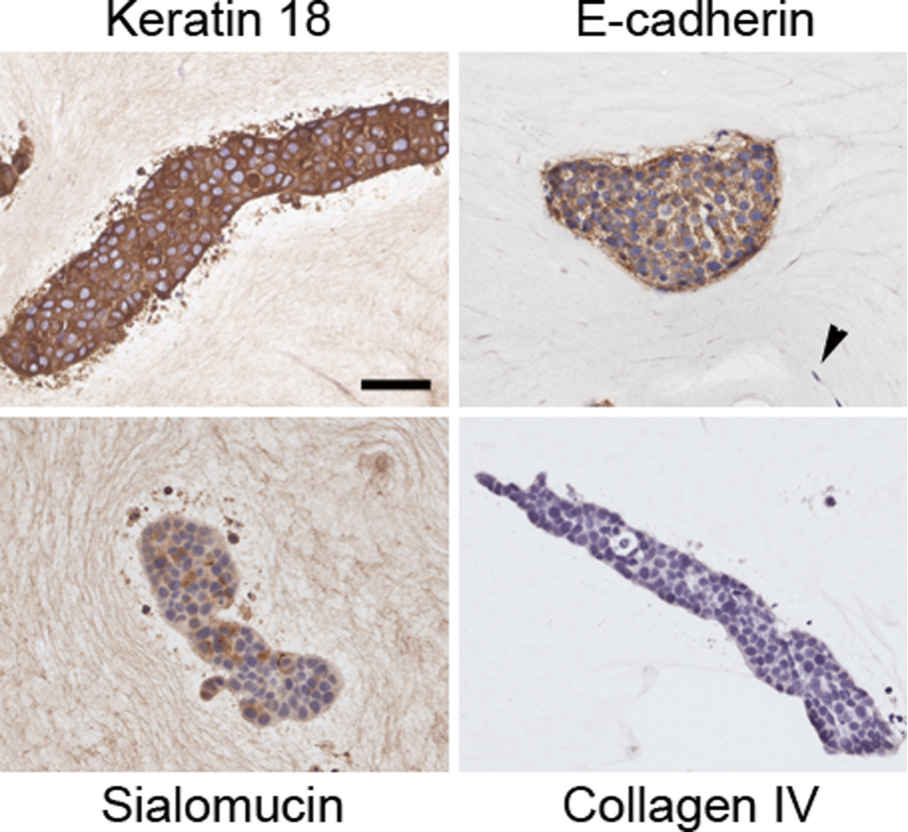
**Characterization of epithelial structures in collagen gels after 2 weeks in culture**. Co-cultures of MCF7 cells and RMF were characterized using immunohistochemical analyses for luminal epithelial cell origin (Keratin 18), adherens junctions (E-cadherin), and polarization (Sialomucin). No staining was observed for the basement membrane marker type IV collagen. Arrowhead points to fibroblast. Scale bar: 50 μm.

**Figure 5:**
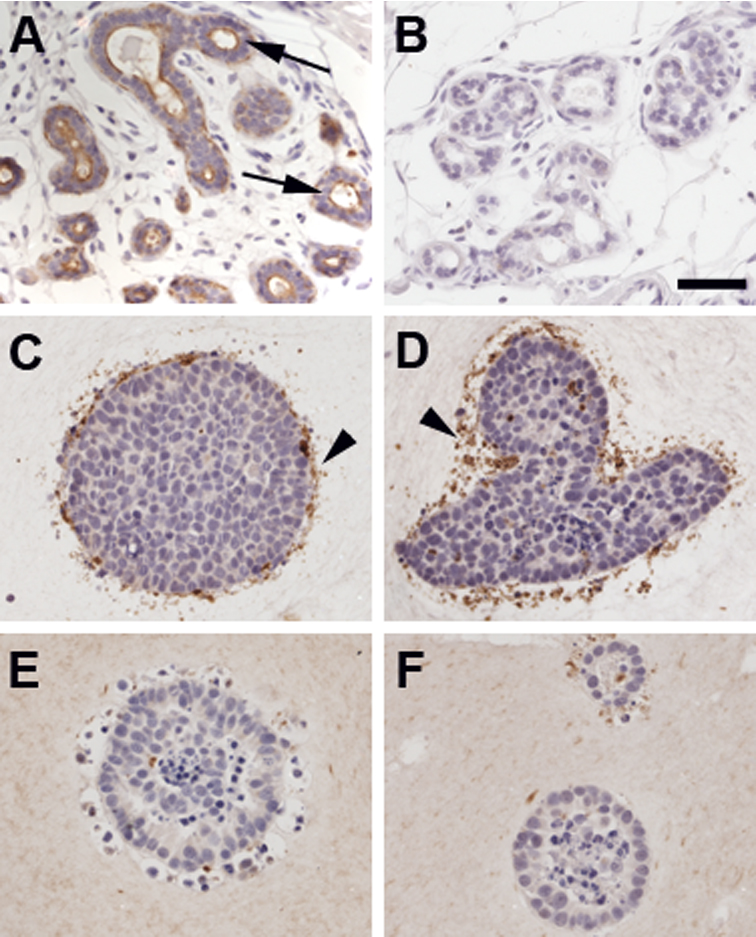
**Immunohistochemical analysis of CEACAM protein expression**. Panel A: representative field of normal human breast showing positive staining for CEACAM in the apical side of the epithelial cells (arrows). Panel B: human breast section used as negative control for antibody specificity. Panels C and D are representative images of MCF7 cells cultured in collagen gels either alone (C) or with RMF (D). Panels E and F represent MCF7 cells cultured in mixed gels either alone (E) or with RMF (F). CEACAM staining was negative in MCF7 cells, regardless of the culture conditions. Arrowheads depict unspecific staining. Scale bar: 50 μm

All MCF7 cells grown alone or in co-culture with RMF in the 3D collagen gels showed positive keratin 18 staining confirming the luminal epithelial cell character of those cells (Figure 4). The lack of smooth muscle actin (SMA) staining provided further evidence that MCF7 cells did not differentiate into myoepithelial cells in these conditions (data not shown). After two weeks in culture, E-cadherin, a marker for cell-cell junctions, showed a positive staining in the MCF7 cells both when grown alone and in co-culture with RMF (Figure 4). Sialomucin, a protein secreted on the apical side of polarized cells, was present in a diffuse pattern after two weeks in culture indicating an incomplete polarization, regardless of the presence of RMF. Furthermore, the absence of type IV collagen staining indicated that the MCF7 cells did not generate a basement membrane (Figure 4). This was further confirmed by the lack of laminin 5 staining, and by the absence of a basement membrane when analyzed using transmission electron microscopy (TEM) (data not shown). It has been reported that the cell-cell adhesion molecule CEACAM1 is necessary for apoptosis to occur and for lumen to form in a 3D rBM model [34]. Even though lumen formation was observed in our model in co-cultures of MCF7 cells and RMF, expression of CEACAM1 was not observed in the MCF7 clusters regardless of the presence or absence of RMF (Figure 5).

### Breast morphogenesis in mixed Matrigel™-collagen gels

#### MCF7 cells in mixed gels

**Figure 6:**
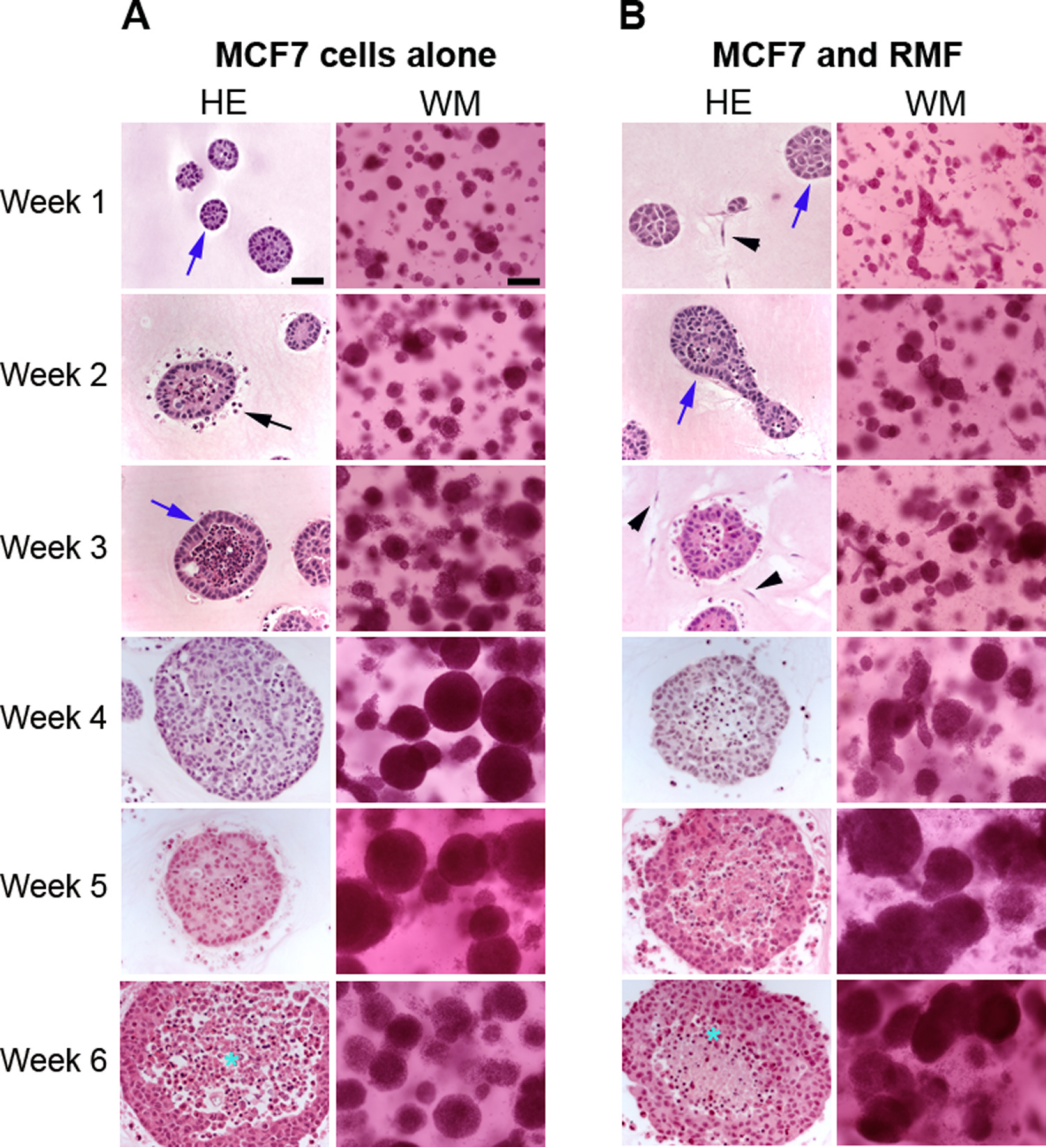
**Epithelial structures observed in mixed Matrigel™-collagen gels**. A) H&E and WM representative of MCF7 cells grown alone in mixed gels for 1-6 weeks. Panel B) H&E and WM of MCF7 cells grown in co-culture with RMF in mixed gels. Black arrows point at dead cells and blue arrows point to polarized cells, arrowheads point to fibroblasts, * indicates dead cells. Scale bars: H&E = 50 μm, WM = 100 μm.

A mixture of Matrigel™ and type I collagen in a volume ratio of 1:1 (final collagen concentration: 1mg/ml) was used to explore whether the addition of rBM, i.e. Matrigel™, to type I collagen modified the MCF7 cells' phenotype. After one week in culture, the MCF7 cells greatly improved their survival in these mixed gels (Figure 6) compared to the same cells grown in type I collagen. Remarkably, starting at one week in culture, polarized alveolar structures were observed (Figure 6); in contrast to the many MCF7 cells that underwent apoptosis in type I collagen gels (Figures 1, 2). The epithelial structures progressively increased in size (Figure 3A), and there was a significant difference in size throughout the three weeks in culture that were quantified (p < 0.05). They also continued to grow through week six. The percentage of cells expressing Ki67 was similar (p > 0.05) between weeks one, two and three with means of 3.83% (3.15-5.34%), 3.93% (2.14-7.16%), and 3.79% (2.92-6.14%), respectively (Figure 3B). Some of the clusters contained dead cells in the periphery after three weeks in culture, and concomitantly, most epithelial clusters were surrounded by a "halo" containing dead cells (Figures 2B, 6A). Importantly, beginning at three weeks substantial cell death was also observed in the center (Figure 2B), suggesting the formation of lumen.

#### Co-culture of MCF7 cells with RMF in mixed gels

**Figure 7:**
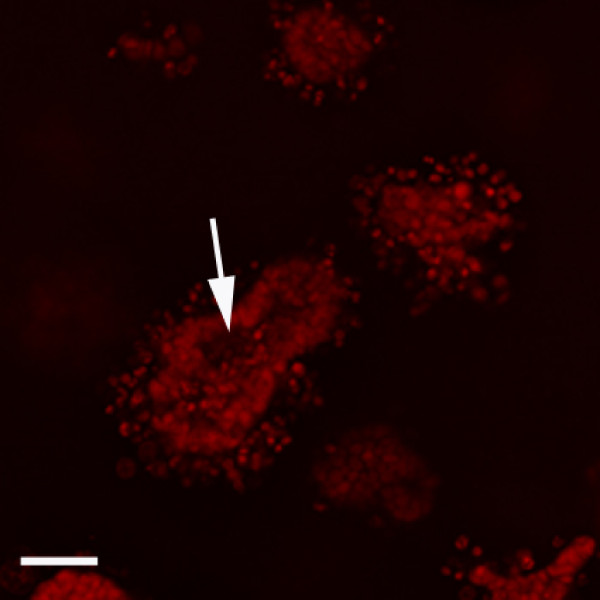
**Presence of lumen in epithelial structures in mixed Matrigel™-collagen gels**. Representative confocal image taken from a whole-mounted gel stained with Carmine-alum of MCF7 cells in co-culture with RMF after 3 weeks in culture. Arrow signals lumen containing some apoptotic cells. Scale bar: 50 μm.

After one week in culture, MCF7 cells co-cultured with RMF formed similar epithelial structures to those observed when MCF cells were grown alone. The area of the epithelial structures was similar to those formed by MCF7 cells grown alone (Figure 3A); however, in contrast to what was observed in the mono-cultures, there was no statistically significant difference over time (p > 0.05). It is worth noting that the epithelial structures continued to grow through week six in culture. The mean percentages of Ki67 positive cells were 2.84% (2.7-2.96%), 3.39% (2.09-6.34%) and 3.37% (2.74-4.52%). Compared to MCF7 cells grown alone in the mixed gels, the co-cultures with RMF had a significantly lower percentage of proliferating epithelial cells after one week (p < 0.05). Furthermore, cells grown in mixed gels had significantly lower proliferation at one and two weeks in culture compared to the same cells grown in type I collagen (p < 0.05); this observation was valid for mono- and co-cultures (Figure 3B). Confocal microscopy revealed that both alveolar- and duct-like structures underwent lumen formation; some dead cells still persisted in the hollowed center and a lumen was observed after two weeks in culture (Figure 7).

#### Characterization of epithelial structures

**Figure 8:**
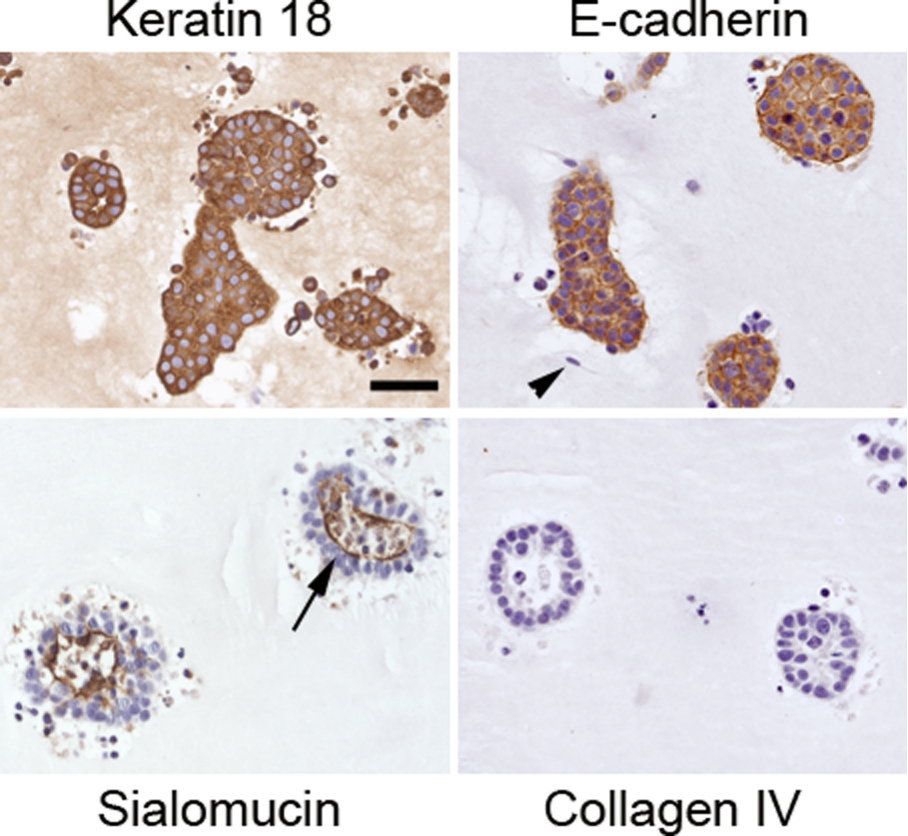
**Characterization of epithelial structures in mixed Matrigel™-collagen gels after two weeks in culture**. Co-cultures of MCF7 cells and RMF were characterized using immunohistochemical analyses for luminal epithelial cell origin (Keratin 18), adherens junctions (E-cadherin), and polarization (Sialomucin). No staining was observed for the basement membrane marker type IV collagen. Arrow points to the luminal localization of Sialomucin staining; arrowhead signals fibroblast. Scale bar: 50 μm.

All MCF7 cells grown either alone or in co-culture with RMF in the mixed gels showed positive keratin 18 staining confirming their luminal epithelial cell origin (Figure 8). In addition, these cells were negative for SMA staining, implying that no myoepithelial cells were present (data not shown). After two weeks in culture, E-cadherin showed a clear basolateral staining pattern regardless of whether MCF7 cells were grown alone or in co-culture with RMF (Figure 8). Furthermore, the sialomucin staining showed apical expression facing the lumen in most epithelial structures indicating that the MCF7 cells were completely polarized. Although the MCF7 cells were able to polarize, a basement membrane was not observed using either anti-type IV collagen (Figure 8) or anti-laminin 5 antibodies (data not shown). These characteristics persisted throughout the length of the experiment. Even though lumen formed, no CEACAM1 staining was observed in the epithelial structures regardless of whether or not RMF were present in the culture (Figure 5).

## Discussion

Understanding normal development and carcinogenesis of the breast requires a multidisciplinary approach because the continuous cross-talk between the tissue compartments, *i.e. *stroma and epithelium, involves both biochemical and biomechanical cues. In an effort to understand some of these interactions and how they modulate the epithelial phenotype, we have further characterized an *in vitro *3D breast cancer model involving the co-culture of MCF7 cells, a widely used human breast cancer cell line, and primary human breast fibroblasts grown within two different extracellular matrices. This promising model revealed that both the extracellular matrix composition and the presence of fibroblasts determined the fate of the epithelial cells and their phenotype. These findings are comparable to those described for the non-tumorigenic breast epithelial MCF10A cells grown in similar conditions [32]. The addition of stromal cells, the utilization of extracellular matrices that more closely resemble the breast tissue microenvironment, and the long-term cultures have revealed the remarkable plasticity of the MCF7 cells under different experimental conditions. Our findings show that MCF7 cells are able 1) to reverse their tumor phenotype almost completely, and 2) to continue growing into large masses that resemble tumors depending on the environment surrounding them and the length of the experiment. This plasticity is precisely the reason why data gathered from 3D culture models ought to be interpreted in the context of each experimental design.

In regards to the reversion of the tumor phenotype, the formation of lumen in breast epithelial structures is one of the hallmarks of the normal mammary phenotype. *In vivo*, the fetal mammary epithelial cords form a lumen at the same time as the mesenchymal cells undergo differentiation [35]. Accordingly, the bi-directional biochemical and biomechanical signals are highly regulated. Lumen formation (associated with a well-differentiated phenotype) and lumen filling (associated with a tumor phenotype) were described in various 3D breast cell culture models; both phenomena are commonly explained as a result of differential gene expression in the epithelial cells. For instance, the pro-apoptotic factor BIM correlates with lumen formation by inducing apoptosis in MCF10A cells [36]. Similarly, MCF10A cells filled the lumen when transfected with ERBB2, CSF-R1 or v-SRC genes [36-38]. Regarding MCF7 cells, the cell-cell adhesion molecule CEACAM1-4 S, which is not expressed in MCF7 cells, is reported to be necessary for apoptosis to occur and lumen to form in a rBM-based 3D culture [34].

Little attention has been given to the role of the extracellular matrix composition and/or the presence of stromal cells in modulating lumen formation in 3D cultures. Here, we showed that MCF7 cells organized into structures containing a lumen in the presence of RMF and/or rBM, and in the absence of CEACAM1 expression. Furthermore, in the mixed gels, apoptosis was observed in the center of the cell clusters both when MCF7 cells were grown alone and in co-culture with RMF. This is a remarkable phenomenon in breast cancer cells grown in 3D conditions and in collagen-based matrices. Interestingly, lumen formation has not been mentioned in studies reporting phenotypic reversion of cancer cells [39]. The only instance in which MCF7 cells did not form a lumen was when the cells were cultured alone in type I collagen matrices. In all cases in which a lumen was observed, apoptosis in the center of the epithelial structures was accompanied by cell polarization, indicating that the latter may be required prior to lumen formation. This is also the case in the developing breast and in the non-tumorigenic breast MCF10A cells grown in 3D cultures [32,40]. Our findings suggest that complex interactions of the matrix components with the epithelial cells are necessary to induce lumen formation, and that such phenomena are not restricted to normal epithelial cells. This correlates well with a report showing that non-tumorigenic mammary epithelial cells filled the lumen and continued to proliferate as a result of changing only the compliance of a 3D collagen matrix, another example of the multifaceted epithelial-stromal crosstalk [30].

Another phenotypic feature of reversion is a reduced proliferation rate. We have observed that the percentage of cells expressing Ki67 decreased over time in type I collagen gels. In addition, the proliferation index was significantly lower in the epithelial structures formed in the mixed gels compared to those formed in the collagen gels after one and two weeks; this correlates well with the hypothesis that cellular organization into defined structures such as alveoli and ducts is accompanied by a decrease in proliferation. It has been reported that normal breast epithelial cells significantly reduced their proliferation rate after polarized alveoli and ducts are formed in 3D cultures [32,41] whereas primary breast carcinoma cells and tumorigenic breast cell lines formed large, disorganized and non-polarized colonies that failed to undergo growth arrest [41]. These observations are consistent with our findings that MCF7 cells in mixed gels undergo near complete phenotypic reversion.

Previously, we reported that RMF co-cultured with the non-tumorigenic human breast MCF10A cells accelerated the initial formation of epithelial structures in collagen gels [32]. Similarly, we now show that RMF provided a "survival" signal and considerably improved the organization of MCF7 cells cultured in the same matrix. Moreover, RMF induced the formation of both round and elongated structures containing polarized cells and lumen. In contrast, many MCF7 cells grown alone in the same collagen matrix underwent apoptosis, and after a lag period, the surviving cells only formed round, disorganized cell clusters. It is worth mentioning that these structures evolved throughout the six week incubation period and that the phenotype of the tissue structures, and the conclusions drawn from these experiments, would have varied had the cultures been stopped at the usual two weeks or earlier [18,19,42].

The addition of Matrigel™ to the collagen gels provided additional evidence for the role of the ECM proteins in modulating the epithelial phenotype. The morphology of the structures formed by the MCF7 cells cultured in mixed gels containing collagen and rBM proteins was different from that observed in collagen gels regardless of whether or not RMF were present in the culture. After one and two weeks in culture, MCF7 cells grown alone already formed alveolar structures containing polarized cells and lumen, indicating that rBM provided "survival" and patterning signal(s) for the MCF7 cells. In the presence of RMF in these gels, MCF7 cells formed both alveolar- and duct-like structures consisting of polarized cells.

In an extensive report, Kenny and colleagues studied the growth of different breast cancer cell lines in an rBM-based 3D culture. These researchers classified the cell lines into four categories depending on their epithelial morphology. MCF7 cells were part of the "mass class", meaning that the cells formed round structures with disorganized nuclei and no lumen [18], which appears comparable to what we observed when MCF7 were grown in collagen gels. However, our findings that MCF7 cells growing alone or in co-culture with RMF were able to organize and polarize in mixed gels indicate that a matrix containing both type I collagen and rBM provided a more advantageous environment for the epithelial cells to differentiate. In addition, because the experiments described by Kenny *et al*. only lasted four days [18], it is difficult to compare our respective data considering the much longer length of our experiments (*i.e. *up to 6 weeks).

It has been previously reported that tumor cells can reverse their malignant phenotype to a normal one both in 3D cultures and *in vivo *[7,39,43,44]. The phenotype reversion in rBM 3D cultures has been defined by the presence of small, growth arrested, well-differentiated and polarized acinar structures [39]. It is worth noting that lumen formation was not included as one of the features of reversion. Using the same model, it has been shown that reversion occurs through modulating cell-adhesion proteins such as beta1 and alpha2 integrins, dystroglycan1, CEACAM1, gap junctions, and EGFR that are aberrantly expressed in tumors cells [19,45-47], and inhibition of PI3K and MAPK pathways [39]. Our results suggest that changes in either matrix composition alone and/or the addition of stromal cells are sufficient to lead to a reversed phenotype that was near normal, *i.e. *organized and polarized epithelial structures, lumen formation, and low proliferation index. In our model, in the presence of RMF, MCF7 cells formed organized acini containing polarized cells and lumen, as well as elongated structures with features that resembled ducts. However, it should also be noted that in all these conditions MCF7 cells were unable to secrete their own basement membrane proteins indicating that the reversal was incomplete.

Despite dramatic changes in epithelial cell morphology due to the presence of RMF and/or different matrices, the continuous, although low, proliferation of the MCF7 cells observed during the six weeks in culture suggests that such a microenvironment was insufficient to completely reverse the MCF7 cell tumor phenotype throughout the length of the experiment, and that more research is necessary to understand how cell proliferation is controlled under these conditions. In order to observe a complete reversion as has been shown with cancer cells *in vivo *[7,48,49], this and other 3D culture models have the potential to be improved by increasing their complexity while adding either different types of stromal cells, various extracellular matrix components and/or modifying the biomechanical properties of the gels. Taking into consideration this and other reports, it becomes apparent that the fate of normal and neoplastic epithelial cells and their organizational phenotype is susceptible of being modified by manipulating a wide array of cellular and extracellular parameters.

## Conclusions

The *in vitro *3D culture models presented herein contribute to a better understanding of the initial steps in tumor formation, tumor phenotype reversion, and are suitable for long-term studies as the cells can be maintained for up to 6 weeks in culture. We have also provided evidence that stromal cells such as fibroblasts are essential for individual and collective cell survival, and for the shape of the MCF7 cells structures. In addition, our results indicate that a matrix containing both type I collagen and rBM provides the right conditions to study tumor phenotype reversion. Finally, it is increasingly apparent that stromal-epithelial interactions can be systematically explored in 3D culture conditions. These tools provide useful surrogate models to deepen our understanding of the role of the biochemical and biomechanical components of the breast during normalcy, carcinogenesis and cancer reversal.

## Abbreviations

The following abbreviations are used in this paper.

3D: 3 Dimensional; DMEM: Dulbecco's modified Eagle's medium; ECM: extracellular matrix; FBS: Fetal bovine serum; MCF7: human breast cancer cell line; MMP3: matrix metalloproteinase 3; rBM: reconstituted basement membrane; RMF: reduction mammoplasty fibroblasts; TEM: transmission electron microscopy

## Competing interests

The authors declare that they have no competing interests.

## Authors' contributions

SK performed all the experiments and analysis and drafted the manuscript. MVM participated in the study design, histological analysis and co-wrote the manuscript with SK. AMS and CS participated in the study coordination, design and interpretation of results and revised the manuscript. All authors read and approved the final manuscript.

## Pre-publication history

The pre-publication history for this paper can be accessed here:

http://www.biomedcentral.com/1471-2407/10/263/prepub
